# Resistive Switching Characteristics of HfO_2_ Thin Films on Mica Substrates Prepared by Sol-Gel Process

**DOI:** 10.3390/nano9081124

**Published:** 2019-08-04

**Authors:** Chao-Feng Liu, Xin-Gui Tang, Lun-Quan Wang, Hui Tang, Yan-Ping Jiang, Qiu-Xiang Liu, Wen-Hua Li, Zhen-Hua Tang

**Affiliations:** School of Physics & Optoelectric Engineering, Guangdong University of Technology, Guangzhou Higher Education Mega Center, Guangzhou 510006, China

**Keywords:** resistance switching, high/low resistance, oxygen defect, conduction mechanism

## Abstract

The resistive switching (RS) characteristics of flexible films deposited on mica substrates have rarely been reported upon, especially flexible HfO_2_ films. A novel flexible Au/HfO_2_/Pt/mica resistive random access memory device was prepared by a sol-gel process, and a Au/HfO_2_/Pt/Ti/SiO_2_/Si (100) device was also prepared for comparison. The HfO_2_ thin films were grown into the monoclinic phase by the proper annealing process at 700 °C, demonstrated by grazing-incidence X-ray diffraction patterns. The ratio of high/low resistance (off/on) reached 1000 and 50 for the two devices, respectively, being relatively stable for the former but not for the latter. The great difference in ratios for the two devices may have been caused by different concentrations of the oxygen defect obtained by the X-ray photoelectron spectroscopy spectra indicating composition and chemical state of the HfO_2_ thin films. The conduction mechanism was dominated by Ohm’s law in the low resistance state, while in high resistance state, Ohmic conduction, space charge limited conduction (SCLC), and trap-filled SCLC conducted together.

## 1. Introduction

Resistive random access memory (RRAM) is a kind of memory in which, according to the different voltage applied to the metal oxide, the resistance of the material changes correspondingly between the high resistance state (HRS) and the low resistance state (LRS), so as to open or block the current flow channel and use this property to store various information [1]. RRAM can significantly increase durability and data transmission speed compared with flash memory devices. The main factor affecting the performance of RRAM is the RS layer, and the performance of different RS layers varies greatly. A variety of materials can be applied as the resistive switching layers of RRAM, such as HfO_2_, SnWO_4_, ZrO_2_, and CuO [2,3,4,5,6], among which binary metal oxides like HfO_2_ are widely regarded as the most promising resistive switching layer [1,7]. The conduction mechanisms of RRAM have been studied in depth, among which Ohmic conduction, Schottky emission, space-charge-limited conduction (SCLC), and trap-assisted tunneling are the most popular [1,8,9,10,11]. The conductive filament (CF) model has also been one of the most recognized models [8]. With the development of science and technology, flexible memory has also been extensively studied in the past decade [12,13]. Due to the advantages of their being inexpensive and lightweight, flexible memristors are more widely used than non-flexible devices such as disposable sensors [14] or indenofluorene-based monomers [15].

Although flexible electronic devices have promising applications in wearable devices, few papers have reported on the RS characteristics of flexible films deposited on mica substrates [16,17,18]. Mica substrates are cheap, easy to prepare, and satisfy the demands of industrial production, which makes them an excellent candidate for preparing flexible RRAM substrates. In this paper, HfO_2_ thin films were grown on flexible mica substrates by the sol-gel method. For comparison of different substrates, HfO_2_ films were also deposited on Pt/Ti/SiO_2_/Si (100) substrates. As a kind of ordinary semiconductor compound, HfO_2_ film has a high dielectric constant and desirable light transmittance with a simple preparation [19]. Due to its thermal stability and excellent retention performance [2,20,21,22], HfO_2_ has been widely studied in the field of RRAM in recent years [23], and is one of the most promising candidates for the resistive switching layer. The results show that the ratio of HRS to LRS exceeded 100 in the HfO_2_-based-non-flexible structure, with excellent stability. In contrast to non-flexible resistive switching, the HfO_2_-based flexible structure demonstrated a pretty good resistive switching characteristic, but its endurance was inferior to non-flexible resistive switching. This HfO_2_-based flexible device has a simple preparation method (sol-gel), inexpensive cost, and excellent flexibility not existing in an HfO_2_-based-non-flexible structure, which conforms to the developing requirements of our time for flexible RRAM.

## 2. Materials and Methods

**Figure 1:**
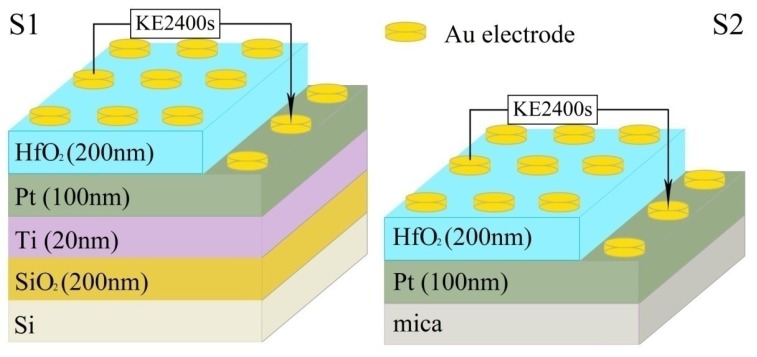
Schematic patterns of the HfO_2_/Pt/Ti/SiO_2_/Si (S1) and HfO_2_/Pt/mica (S2) devices.

Using the sol-gel method for coating, a certain amount of hafnium acetone was weighed as the raw material, the magnetic stirrer was used to dissolve it in acetic acid until a colloid formed, the hafnium acetone colloid was spirally coated onto the different substrate by a rotary coating machine and then placed on a drying platform. The drying platform was heated from room temperature to 300 °C for 10 min, which decomposed hafnium acetone into HfO_2_ at high temperature. In this paper, there were two samples of different substrates, HfO_2_/Pt/Ti/SiO_2_/Si and HfO_2_/Pt/mica flexible structures. For further discussion, the structures of HfO_2_/Pt/Ti/SiO_2_/Si and HfO_2_/Pt/mica are abbreviated as S1 and S2, respectively, as shown in Figure 1. Both S1 and S2 were annealed at 700 °C in air atmosphere for 30 min. After annealing, an Au point electrode with diameter of 0.5 mm was plated on the sample using a small high-vacuum coating machine and a mask template with diameter of 0.5 mm at room temperature for two min to form a top–bottom (TB) electrode structure.

Current–voltage (*I-V*) and endurance characteristics were measured by the Keithley 2400 s instrument. Atomic force microscopy (AFM) showed the surface morphology of the film, and field emission scanning electron microscopy (FESEM) could clearly observe the thickness of the HfO_2_ thin film and the layers between substrate and film. Additionally, the phase structures of HfO_2_ films were analyzed by grazing-incidence X-ray diffraction (GIXRD) with an incident angle of 1°. Moreover, X-ray photoelectron spectroscopy (XPS) analyses of the HfO_2_ thin films were carried out using an Escalab 250Xi X-ray photoelectron spectrometer.

## 3. Results and Discussion

**Figure 2:**
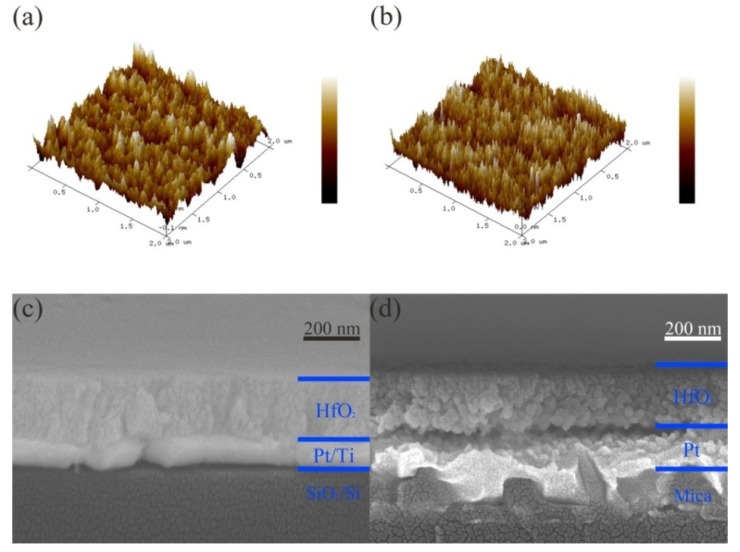
(**a**,**b**) Atomic force microscopy patterns of HfO_2_ thin films in S1 and S2 devices, respectively; (**c**,**d**) typical cross-sectional scanning electron microscope images of S1 and S2, respectively.

**Figure 3:**
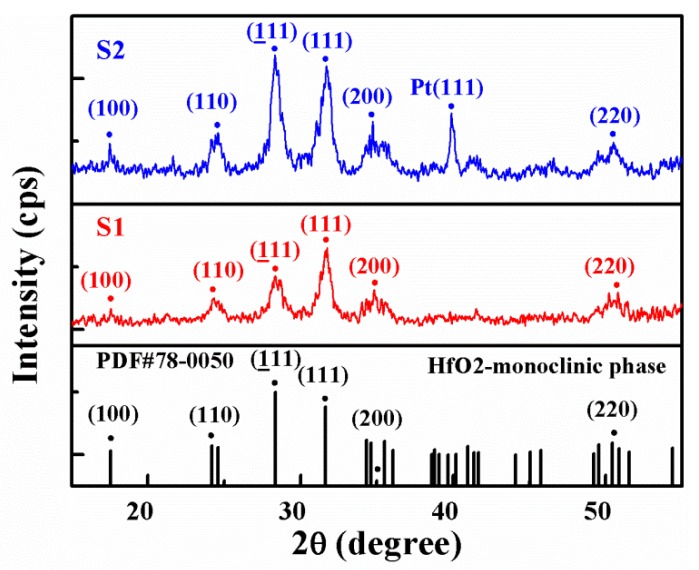
Grazing-incidence X-ray diffraction patterns of HfO_2_ thin films in S1 and S2.

It can be seen from Figure 2a,b that the grain size of the HfO_2_ thin films after annealing was relatively small, which was due to the low annealing temperature and short annealing time. The SEM cross-sectional views of S1 and S2 show a dense layer of HfO_2_ with a thickness of ~200 nm, and a dense Pt layer with a thickness of ~100 nm can be seen in all cases, as shown in Figure 2c,d. Additionally, the density and adhesion of HfO_2_ on a typical Pt substrate were better than that on a flexible substrate. Figure 3 indicates the GIXRD patterns of the HfO_2_ films grown on two different devices. As can be seen from Figure 3, the HfO_2_ thin films had high crystallinity—a polycrystalline (100), (110), (111), (111), (200), and (220) oriented monoclinic phase structure [24,25]. Additionally, the PDF#78-0050 of the HfO_2_ monoclinic phase is inserted in Figure 3 to better identify the XRD peak of the HfO_2_ film. HfO_2_ with a monoclinic phase structure can accumulate oxygen vacancies [26]. The relatively small GIXRD peak intensity shows the smaller grain size of the HfO_2_ thin films, corresponding to the results of the SEM and AFM analyses. Additionally, a Pt (111) oriented peak existed in the S1 device.

**Figure 4:**
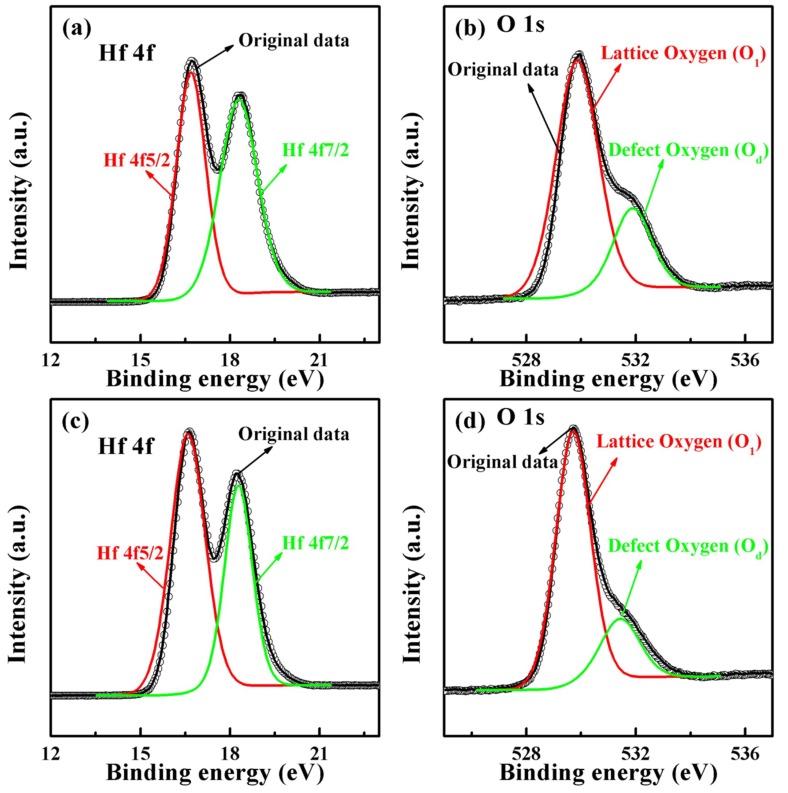
(**a**,**b**) The X-ray photoelectron spectroscopy (XPS) spectra of the S1 device; (**c**,**d**) the XPS spectra of the S2 device; (**b**,**d**) show the different oxygen intensities after fitting the peak.

As shown in Figure 4a,c, Hf 4f core levels of HfO_2_ thin films layers in all cases were deconvoluted into two Gaussian peaks (16.7 eV for Hf 4f5/2 and 18.3 eV for Hf 4f7/2, indicated by the red line and green line, respectively) [27,28,29]. Figure 4b,d shows XPS spectra of the O 1 s core levels of the HfO_2_ thin films layers in all cases. Obviously, the Gaussian peak with a binding energy of 529.7eV was defined as lattice oxygen (O_l_), corresponding to the oxygen in the HfO_2_ matrix; the other, with a binding energy of 531.5eV, was defined as defect oxygen (O_d_), caused by the defects of oxygen vacancies in the HfO_2_ thin film layers. Previous research has indicated the higher the intensity of O_d_, the higher the concentration of oxygen vacancy [5]. The ratio of Hf/O_l_ in all devices was ~2, signifying the existence of HfO_2_ [30,31]. Furthermore, the ratio of O_l_/O_d_ in S1 devices (0.32) was larger than that of S2 devices (0.25) and the ratio of O_d_ in S1 devices to that in S2 devices was 0.82, resulting in the difference of HRS/LRS ratio between the two devices, which was consistent with *I-V* characteristics.

**Figure 5:**
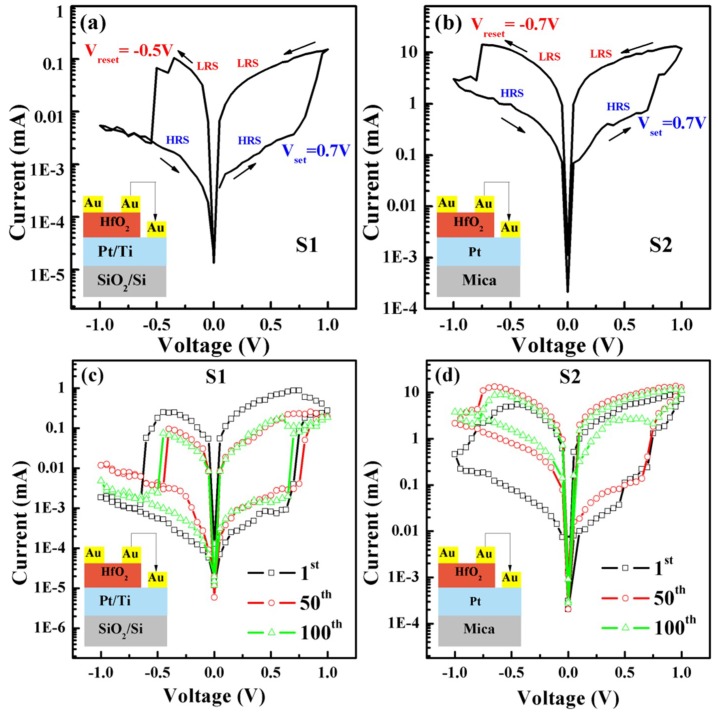
Resistive switching characteristics of (**a**) Au/HfO_2_/Pt/Ti/SiO_2_/Si, (**b**) Au/HfO_2_/Pt/mica, (**c**) Au/HfO_2_/Pt/Ti/SiO_2_/Si with 100 sweep cycles, and (**d**) Au/HfO_2_/Pt/mica with 100 sweep cycles.

**Figure 6:**
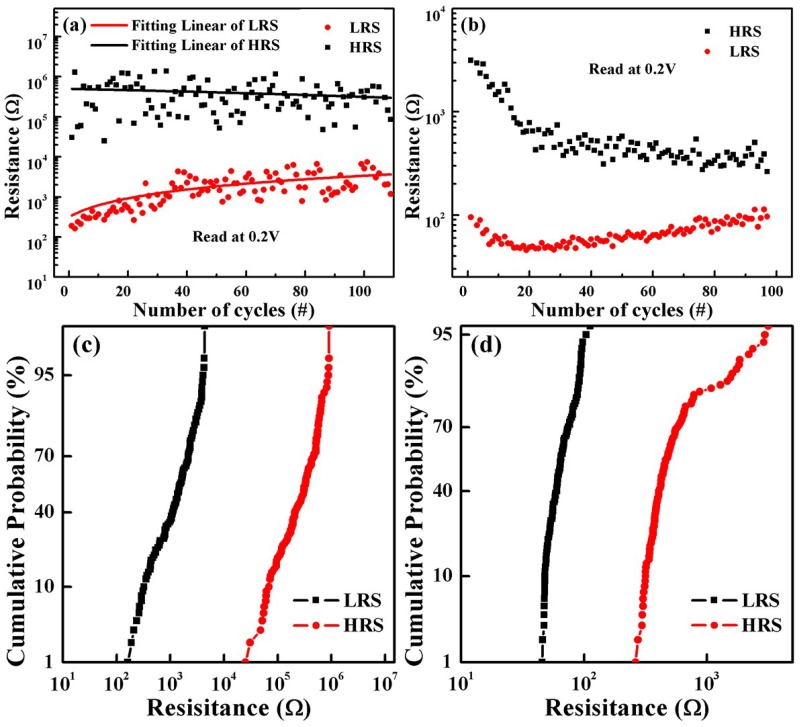
Endurance characteristics of (**a**) Au/HfO_2_/Pt/Ti/SiO_2_/Si and (**b**) Au/HfO_2_/Pt/mica RRAM devices at room temperature; (**c**,**d**) the cumulative probability plots of high resistance state and low resistance state for the two devices, respectively, at a reading voltage of 0.2 V.

Figure 5a,b shows the excellent resistance switching behaviors of the S1 and S2 structures. It is apparent that the V_set_ and V_reset_ of the S1 devices were 0.7 V and −0.5 V respectively, while the V_set_ and V_reset_ of the S2 devices were 0.7 V and −0.7 V respectively [22,32]. In addition, because the grain size of HfO_2_ for S2 is larger than that for S1, based on the FESEM patterns (Figures S2 and S3), the switching currents of the S2 device were much larger than those of the S1 device. When the applied bias increased from 0 V to 0.7 V, both devices remain “off” (HRS). The device will be converted to LRS if the voltage reaches 0.7 V (V_set_). Subsequently, with a voltage loop of 0.7 V to 1 V to −0.7 V for S2 (0.7 V to 1 V to −0.5 V for S1), the device will always stay in “on” (LRS). When the voltage reaches −0.7 V (−0.5 V for S1) for the first time, the device will immediately be reset to “off” (HRS), and remain HRS all the way up to 0 V. The turn-on slope of S1 was calculated as 0.3 V/decade and was almost equal to that of S2, which depicted a switching speed in S2 consistent with S1; the ratio of HRS and LRS for the S1 device (~100) was greater than that of S2 device (~50), which also indicates that the S1 device had better switching characteristics than the S2 device. Additionally, resistive switching characteristics with 100 sweep cycles are depicted in Figure 2c,d. It can be seen clearly that the HRS/LRS ratio of S2 device gradually decreased from the 50th cycle; by contrast, the HRS/LRS ratio of the S1 device was almost stable when a forward bias was applied. From the results above, the device formed on the flexible substrate had the characteristics of typical RRAM. Figure 6 shows a stable resistance state (LRS/HRS) of the S1 device, with a reading voltage of 0.2 V for 100 sweep cycles at room temperature. The fitting linear curves in Figure 6a exhibit a stable off/on ratio for S1 RRAM devices, starting at 1000 times, slowly falling to 100 times, and then leveling off. However, as can be seen from Figure 6b, the S2 devices exhibited poor endurance characteristics, with rapid fatigue from 50 times to 10 times followed by leveling off. For the sake of illustrating the variation in HRS resistance and LRS resistance, Figure 6c,d compares the cumulative probability plots of HRS and LRS for the two devices at a reading voltage of 0.2 V. Compared to the S2 device, the S1 device exhibited a stable distribution of off/on resistance [33]. From the above analysis, the performance of S2 device was not as good as that of the S1device. In order to better illustrate the poor fatigue characteristics of S1 devices, repeatability tests are also conducted, as is shown in Figure S1. This demonstrates the shortcoming of mica-based devices that must be improved upon but cannot be at present. 

**Figure 7:**
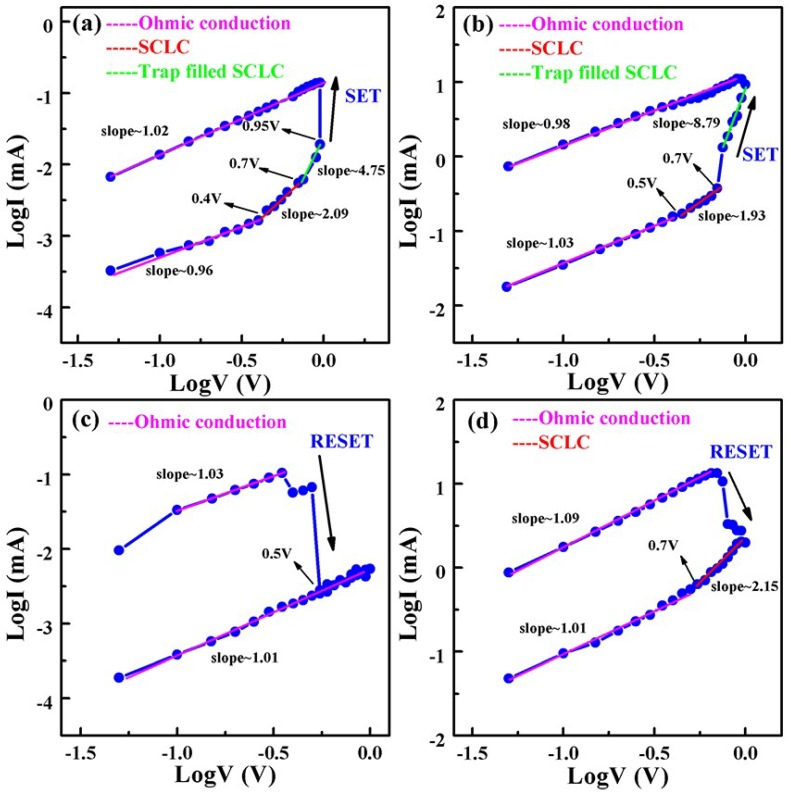
log*I*–log*V* plots in (**a**) Au/HfO_2_/Pt/Ti/SiO_2_/Si and (**b**) Au/HfO_2_/Pt/mica RRAM devices under positive voltage; (**c**) Au/HfO_2_/Pt/Ti/SiO_2_/Si and (**d**) Au/HfO_2_/Pt/mica RRAM devices under negative voltage.

Figure 7 indicates that Ohmic conduction (*I* is proportional to *V*) and SCLC (*I* is proportional to *V*^2^) were the main conduction mechanisms. The current density of SCLC can be depicted as following [1]:

(1) $$J_{SCLC}=\frac{9}{8}\mu \varepsilon \frac{V^{2}}{d^{3}}$$

where *ε* is the permittivity of the film, *μ* is the electron mobility, *V* is the voltage, and *d* is the thickness of the film. Furthermore, it can be reasonably inferred that the conductive mechanism is dominated by trap-filled SCLC (indicated by the green line) when the forward bias is more than 0.7 V. The current density of trap-filled SCLC can be depicted as following [1]:

(2) $$J_{TFSCLC}=q^{1-l}\mu N{\left(\frac{2l+1}{l+1}\right)}^{l+1}{\left(\frac{l}{l+1}\frac{\varepsilon_{r}\varepsilon_{0}}{N_{t}}\right)}^{l}\frac{V^{l+1}}{d^{2l+1}}$$

where *q*, *l*, *μ*, *ε_r_*, *ε*_0_, *N_t_*, *N*, *V*, and *d* are the elemental charge, the ratio of the characteristic temperature of the trap distribution to the operating temperature, the carrier mobility, the permittivity of the film, the permittivity of free space, the trap density, the density of state in the conduction band or valence band, the applied voltage, and the film thickness, respectively.

The log*I* versus log*V* plots have been fitted linearly to analyze the conduction mechanisms of S1 and S2 devices comprehensively. Figure 7a,b exhibits four different slope regions for S1 and S2 devices in positive sweeps, which represent three different conduction mechanisms: Ohmic conduction (slope = 1), SCLC (slope = 2), and trap-filled SCLC (slope > 2). The conduction mechanism of the S1 device was consistent with S2 device, which transferred from Ohmic conduction to SCLC at 0.4 V for the S1 device and 0.5 V for the S2 device, and then to trap-filled SCLC at 0.7 V for all cases. According to the SCLC mechanism, the electron trap is conceived as an oxygen vacancy, and the resistance slowly decreases as the oxygen vacancy filled with electrons, according to Child’s law. However, when the oxygen vacancy is brimming with electrons, the latter will flow past the conduction band, so that the devices will be switched from HRS to LRS [34]. Note that the slope of LRS was almost equal to 1 for all devices, indicating the formation of CF. For the S1 devices in negative sweeps, the Ohmic mechanism ran through the LRS and HRS, as is shown in Figure 7c, while for S2 devices in negative sweeps, it can be clearly observed that the slope was 2.15 for voltage ranges from −1 V to −0.7 V, demonstrating that the CF formed by oxygen vacancies was broken, resulting in reset of resistance state from LRS to HRS. At the same time, the electrons were quickly disengaged from the oxygen vacancy. In conclusion, the conduction mechanism was dominated by Ohmic conduction in LRS, while in HRS, the Ohmic conduction and SCLC conducted together.

**Figure 8:**
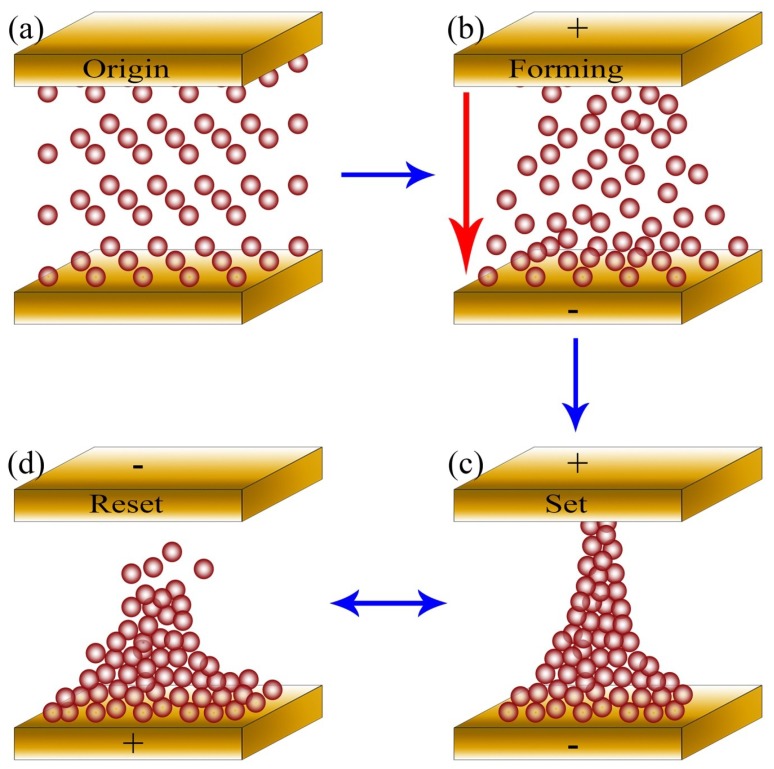
Schematic diagram explaining the conduction mechanism: (**a**) The RS is very high because the device does not form CF; (**b**) When a positive bias is applied, the oxygen vacancies move towards the negative electrode and a CF is formed; (**c**) The device is in the SET state because the oxygen vacancies has formed CF; (**d**) When the voltage is reversed, the CF immediately rupture.

According to the analysis of XPS spectra and conduction mechanism, the CF caused by oxygen vacancy dominated the resistance switching mechanism [5,35]. As shown in Figure 8, a typical CF model has been proposed to better illustrate the influence of O_d_. A large number of defects caused by oxygen vacancies exist in HfO_2_ thin film layers, distributing randomly in the thin film layer and the interface layer without biased voltage, corresponding to the HRS depicted in Figure 8a, which is consistent with the HRS at zero voltage shown in Figure 5a,b. When a forward bias (<0.4 V for S1 devices, <0.5 V for S2 devices) was applied to the device, the conduction mechanism obeyed Ohm’s law. The trap was gradually filled by injected electrons as the applied voltage increased (0.4 V–0.7 V for S1 devices, 0.5–0.7 V for S2 devices), the CF formed, as shown in Figure 8b, and the conduction mechanism was dominated by Child’s law (SCLC). At this time, it corresponded to the HRS of the positive bias voltage (0–0.7 V) in Figure 5a,b. Due to the action of the electric field force, the oxygen ions drifted upward and accumulated at one end of the top electrode, forming a conductive bridge via these oxygen vacancies, while the CF built by oxygen vacancies connected the top and bottom electrodes, resulting in the SET process, as shown in Figure 8c [32,36]. It can also be seen from Figure 5a,b that when the forward voltage was greater than 0.7 V for the first time, the CF was formed, and the RS converted from HRS to LRS. When the voltage loop dropped from 1 V to −0.5 V, the RS remained “on” (LRS), as shown in Figure 5a,b, which is consistent with Figure 8c. Meanwhile, the conduction mechanism was controlled by Ohmic conduction for the existence of CF. Figure 8d exhibits that as the reverse bias was applied to the device, the oxygen ions drifted downward and then combined with the oxygen vacancy, resulting in the rupture of the CF. Combined with the analysis in Figure 5a,b, when the reverse bias voltage reached a certain value (−0.5 V for S1, −0.7 V for S2), the CF completely ruptured, resulting in an instant reset from LRS to HRS. Subsequently, the RS was always off (HRS) while the voltage loop went from −0.7 V to −1 V to 0 V for S2 or from −0.5 V to −1 V to 0 V for S1. The formation and rupture of the CF perfectly explains the principle of resistance switching, which is consistent with the conductive mechanism and *I-V* characteristics.

## 4. Conclusions

In summary, an Au/HfO_2_/Pt/Ti/SiO_2_/Si device and an Au/HfO_2_/Pt/mica device were fabricated by the sol-gel method. As a popular research material, the S1 device structure has been thoroughly studied. At present, the breakthrough point was whether the HfO_2_ with a flexible structure would have the same performance as the typical device. Herein, quite a few advantages and disadvantages of flexible HfO_2_ devices have been identified by analyzing the differences between the S1 and S2 devices. The O_d_ intensity of XPS spectra for the S2 device was lower than for the S1 device, which indirectly illustrates that the HRS/LRS ratio of the S2 device was lower. Meanwhile, the *I*-*V* characteristic also demonstrated the difference in off/on ratio. Nevertheless, HRS/LRS ratio of the S2 device also reached 50, which is enough to illustrate the potential application of flexible HfO_2_ device and that they are worth further study. For the Au/HfO_2_/Pt/mica device, the conduction mechanism was dominated by Ohmic conduction in LRS, and Ohmic conduction and SCLC conduction together in HRS. There is no doubt that the CF model can perfectly illustrate this conduction mechanism. The potential problem is the poor fatigue characteristics of the HfO_2_-mica-based RRAM, which cannot be solved at present, but we hope to solve effectively in the future. 

## Supplementary Materials

The following are available online at https://www.mdpi.com/2079-4991/9/8/1124/s1, Figure S1: Current–Voltage plots of repeated samples, Figure S2: The FESEM pattern of the surface for S1 device, Figure S3: The FESEM pattern of the surface for S2 device.
