# Supplementary figures and images for: Resistive Switching Characteristics of HfO2 Thin Films on Mica Substrates Prepared by Sol-Gel Process
Source: Nanomaterials (Basel). 2019 Aug 4;9(8):1124. doi: 10.3390/nano9081124 (PMC6723579; doi:10.3390/nano9081124)

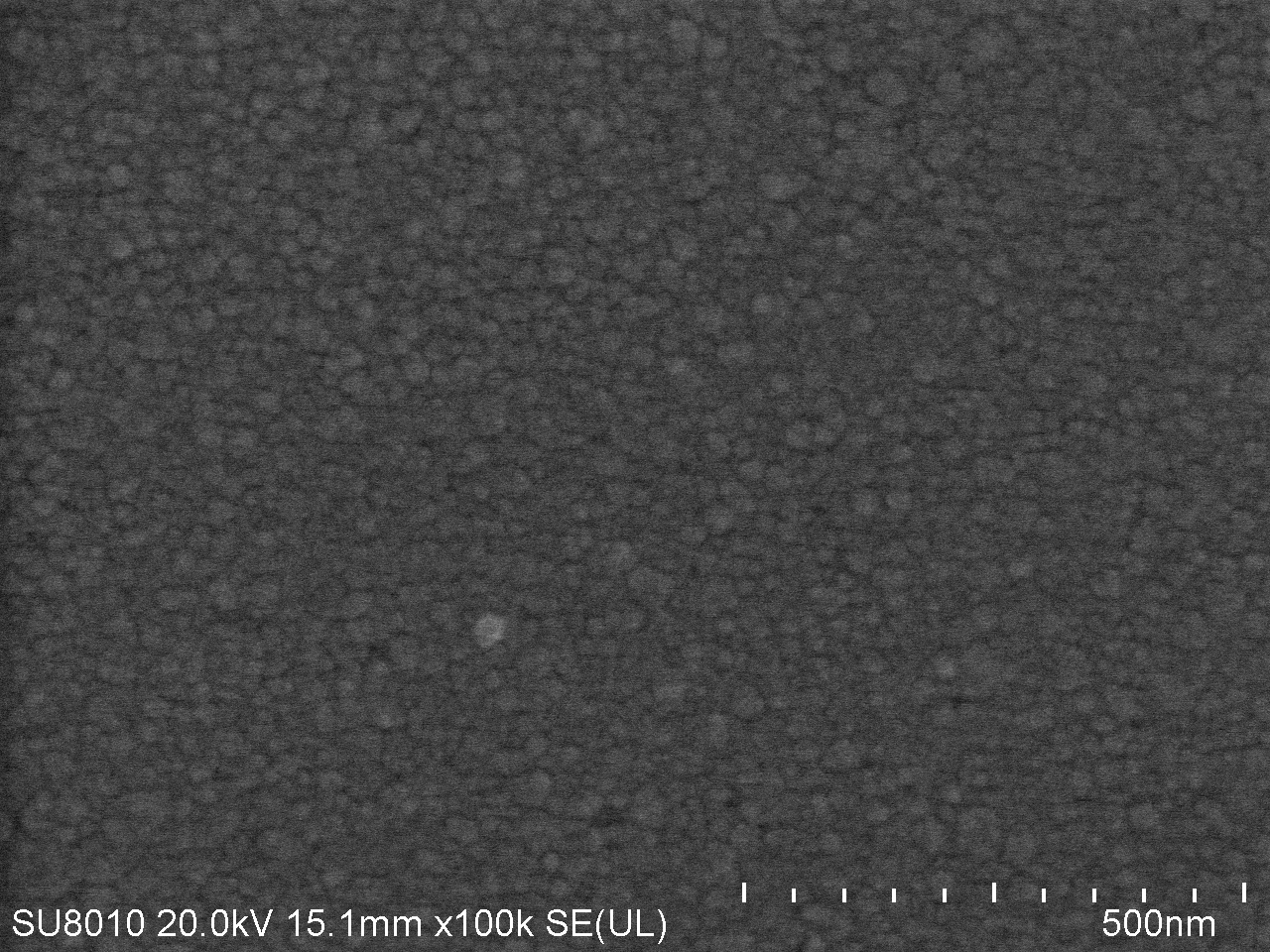

Supplement: Supplementary file 1 [file nanomaterials-09-01124-s001.zip › nanomaterials-561794-supp/Supplementary Materials/Figure S2.tif]

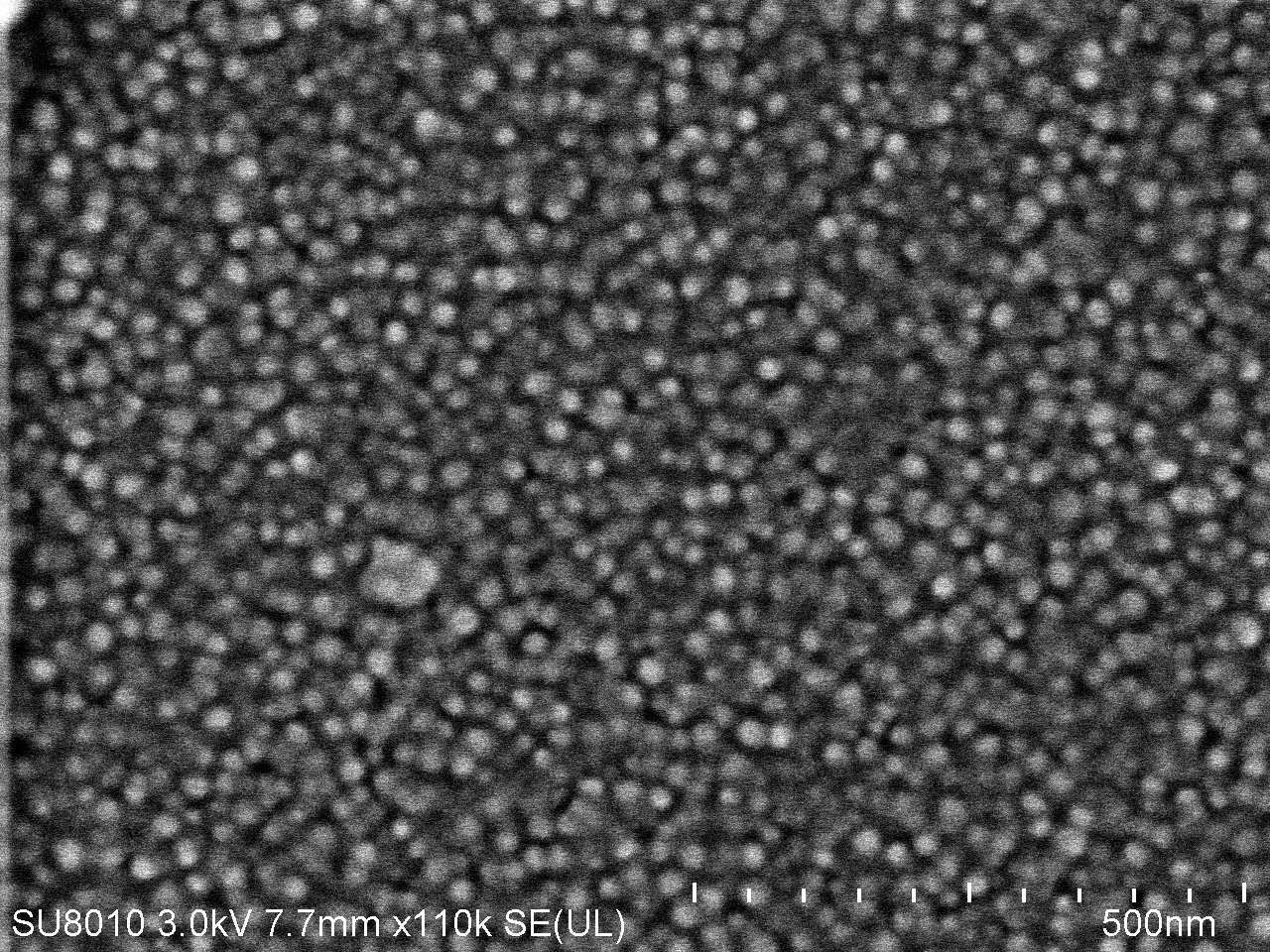

Supplement: Supplementary file 1 [file nanomaterials-09-01124-s001.zip › nanomaterials-561794-supp/Supplementary Materials/Figure S3.tif]
